# Physiological Differences Between Low Versus High Skeletal Muscle Hypertrophic Responders to Resistance Exercise Training: Current Perspectives and Future Research Directions

**DOI:** 10.3389/fphys.2018.00834

**Published:** 2018-07-04

**Authors:** Michael D. Roberts, Cody T. Haun, Christopher B. Mobley, Petey W. Mumford, Matthew A. Romero, Paul A. Roberson, Christopher G. Vann, John J. McCarthy

**Affiliations:** ^1^School of Kinesiology, Auburn University, Auburn, AL, United States; ^2^Department of Physiology, University of Kentucky College of Medicine, Lexington, KY, United States

**Keywords:** hypertrophy, ribosome biogenesis, satellite cells, microRNAs, IGF-1, androgen receptor

## Abstract

Numerous reports suggest there are low and high skeletal muscle hypertrophic responders following weeks to months of structured resistance exercise training (referred to as low and high responders herein). Specifically, divergent alterations in muscle fiber cross sectional area (fCSA), vastus lateralis thickness, and whole body lean tissue mass have been shown to occur in high versus low responders. Differential responses in ribosome biogenesis and subsequent protein synthetic rates during training seemingly explain some of this individual variation in humans, and mechanistic *in vitro* and rodent studies provide further evidence that ribosome biogenesis is critical for muscle hypertrophy. High responders may experience a greater increase in satellite cell proliferation during training versus low responders. This phenomenon could serve to maintain an adequate myonuclear domain size or assist in extracellular remodeling to support myofiber growth. High responders may also express a muscle microRNA profile during training that enhances insulin-like growth factor-1 (IGF-1) mRNA expression, although more studies are needed to better validate this mechanism. Higher intramuscular androgen receptor protein content has been reported in high versus low responders following training, and this mechanism may enhance the hypertrophic effects of testosterone during training. While high responders likely possess “good genetics,” such evidence has been confined to single gene candidates which typically share marginal variance with hypertrophic outcomes following training (e.g., different myostatin and IGF-1 alleles). Limited evidence also suggests pre-training muscle fiber type composition and self-reported dietary habits (e.g., calorie and protein intake) do not differ between high versus low responders. Only a handful of studies have examined muscle biomarkers that are differentially expressed between low versus high responders. Thus, other molecular and physiological variables which could potentially affect the skeletal muscle hypertrophic response to resistance exercise training are also discussed including rDNA copy number, extracellular matrix and connective tissue properties, the inflammatory response to training, and mitochondrial as well as vascular characteristics.

## Introduction

Physiological factors that affect trait responsiveness to exercise training (e.g., changes in aerobic capacity, strength, or muscle growth) have gained widespread research interest. From a historical perspective, this interest was largely inspired by the renowned HERITAGE study whereby individual VO_2_max changes reportedly ranged from almost no gain to a 100% increase following 20 weeks of endurance training in previously sedentary individuals (Bouchard and Rankinen, 2001). Additionally, Van Etten et al. (1994) examined the skeletal muscle hypertrophic response in individuals that were classified as “slender” of “solid” following 12 weeks of resistance exercise training. Subjects were classified on the basis of their fat-free mass index (FFMI) determined by skinfolds, in which slender subjects had comparatively lower values relative to solid subjects. These authors reported solid subjects presented significant increases in fat-free mass following training (+1.6 kg), while slender subjects experienced virtually no gain in fat-free mass. While this paper did not examine potential biomarkers which could have facilitated these divergent responses, the authors did conclude that future research should “*study the mechanism responsible for differences in weight-training-induced changes in fat free mass.*”

More than a decade later Bamman et al. (2007) published a seminal paper reporting that different skeletal muscle biomarkers exist between skeletal muscle hypertrophic response clusters following 16 weeks of training. “Extreme responders” (termed high responders herein) presented robust increases in muscle fiber cross-sectional area (fCSA) relative to “non-responders” (termed low responders herein) following training. High responders also expressed higher levels of skeletal muscle insulin-like growth factor-1 (IGF-1) mRNA variants as well as an mRNA indicative of satellite cell differentiation (myogenin) relative to low responders following training. Similar approaches were subsequently implemented by Bamman’s laboratory (Kim et al., 2007; Petrella et al., 2008; Thalacker-Mercer et al., 2013; Stec et al., 2016), our laboratory (Mobley et al., 2018a), and others (Davidsen et al., 2011; Ogasawara et al., 2016) with the intent of identifying skeletal muscle biomarkers associated with high versus low response clusters following weeks to months of resistance exercise training. The purpose of this review is to summarize these research findings. Given that only a handful of studies have examined differentially expressed muscle biomarkers between low versus high responders, we also propose less examined factors which may contribute to the differential hypertrophic responses that occur during resistance exercise training and should be further investigated.

## A Brief Overview of Mechanisms That Facilitate Hypertrophy in Response to Resistance Exercise Training

Skeletal muscle hypertrophy in response to resistance exercise training is likely influenced through the interaction of numerous extrinsic and intrinsic factors. Indeed, extrinsic factors could (e.g., sleep patterns) or have (e.g., nutrition) been shown to influence intrinsic cellular responses to resistance exercise training, and these topics are discussed in greater detail elsewhere (Campbell et al., 2007; Knowles et al., 2018). However, for the purpose of this review, three key intrinsic factors which have been generally regarded to influence the hypertrophic response to resistance exercise training will be discussed. These intrinsic factors include: (a) an upregulation in myofibrillar and overall muscle protein synthesis (MyoPS and MPS, respectively) during post-exercise periods which is largely modulated through mammalian target of rapamycin complex 1 (mTORC1) signaling, (b) a reduction in skeletal muscle proteolysis during post-exercise periods, and (c) an increase in satellite cell-mediated myonuclear addition.

mTORC1 is a multi-subunit complex that consists of the mTOR protein, Raptor and mTOR associated protein LST8 homolog (mLST8) (Bond, 2016). Active mTORC1 complexes are localized to lysosomes in the cell body (Betz and Hall, 2013), and these complexes possess kinase activity to phosphorylate downstream target proteins that facilitate translation initiation and upregulate MyoPS and MPS (Wang and Proud, 2006). From a mechanistic perspective, mTORC1 activity is critical for resistance exercise-induced increases in MPS and MyoPS. For instance, pharmacological mTORC1 inhibition via rapamycin substantially abrogates post-exercise MPS increases in humans and rodents (Drummond et al., 2009; West et al., 2016). Further, several studies suggest the magnitude increase of mTORC1 signaling and MyoPS following a resistance exercise bout are predictive of longer-term skeletal muscle hypertrophy. Post-exercise increases in MyoPS rates up to 6 h following a naïve training bout have been shown to poorly correlate with quadriceps CSA increases following 16 weeks of subsequent training (Mitchell et al., 2014). However, subsequent studies indicate post-absorptive MyoPS elevations weeks into training are associated with skeletal muscle hypertrophy given that the initial trauma of training (e.g., z-line streaming and heightened proteolysis) likely subsides by these time points (Damas et al., 2016; Reidy et al., 2017a). Additionally, although some equivocal findings exist (Mitchell et al., 2012), several rodent and human studies have reported the post-exercise phosphorylation status of downstream mTORC1 targets (i.e., p70s6k and 4EBP-1) are associated with muscle hypertrophy following chronic resistance exercise training (Baar and Esser, 1999; Terzis et al., 2008; Hulmi et al., 2009; Mayhew et al., 2009; Mitchell et al., 2013, 2014). Bodine et al. (2001) published a landmark report in mice strengthening the evidence that mTORC1 activity is obligatory for overload-induced hypertrophy; specifically, these authors noted synergist ablation-induced plantaris hypertrophy and p70s6k activity was completely abrogated with 7 and 14 days of rapamycin administration. Longer-term post-exercise MyoPS and MPS responses using the orally ingested deuterium oxide (D_2_O) tracer have allowed for the reappraisal of fractional synthesis rates days (rather than hours using infused ^13^C tracers) following a single exercise bout (Wilkinson et al., 2014). Aside from the aforementioned Damas et al. (2016) study suggesting 24 h post-exercise MyoPS elevations occur weeks into training following single exercise bouts, Brook et al. (2015) recently employed the D_2_O tracer on a weekly basis over a 6-week unilateral leg extensor resistance exercise training study to examine longer-term MyoPS responses to training. These authors reported that, relative to the non-trained leg, MyoPS levels were significantly elevated with training from weeks 0 to 3, but not weeks 3 to 6, and this finding associated with diminished mTORC1 signaling following single exercise bouts at weeks 3 and 6 relative to the first bout at week 0. Thus, it is apparent that mTORC1 is a critical signaling node for increasing MyoPS, MPS, and eventual skeletal muscle hypertrophy in response to resistance exercise training in humans and rodents, or overload in rodents. What cannot be discounted, however, is the contribution of other signaling molecules to skeletal muscle hypertrophy [e.g., mTORC-1 independent Yes-Associated Protein (YAP) signaling, p38 MAPK signaling, Wnt/beta-catenin signaling], and these signaling cascades are discussed in greater detail in elsewhere (Armstrong and Esser, 2005; Norrby and Tagerud, 2010; Goodman et al., 2015; Watt et al., 2018).

There are multiple resistance exercise- or overload-responsive mechanisms that up-regulate mTORC1 activity (Hornberger, 2011). For instance, Hornberger et al. (2006) noted synergist ablation in rodents activates a mechano-sensitive signaling cascade to increase intracellular phosphatidic acid levels and activate mTORC1 signaling. Additionally, transmembrane proteins (e.g., integrins) and associated intracellular proteins (e.g., focal adhesion kinase) act to potentially enhance mTORC1 signaling in rodent skeletal and cardiac muscle subjected to acute eccentric loading or overload (Fluck et al., 1999; Lueders et al., 2011; Clemente et al., 2012), as well as in human skeletal muscle following chronic eccentric training (Franchi et al., 2018b). Resistance exercise also up-regulates skeletal muscle IGF-1 transcript variants during post-exercise periods in humans (Hameed et al., 2003; Roberts et al., 2010), and these variants can be encoded into IGF-1 isoforms which function to increase mTORC1 activity through IGF-1 receptor-mediated Akt activation (Rommel et al., 2001; Schiaffino and Mammucari, 2011). Intramuscular PGF_2α_ prostaglandin levels increase following resistance exercise (Trappe et al., 2001), and this signaling mediator has been shown to increase MPS through mTORC1 activation (Markworth and Cameron-Smith, 2011). Myostatin (MSTN) mRNA levels as well as downstream SMAD signaling are also down-regulated in humans following one or multiple resistance exercise bouts (Louis et al., 2007; Dalbo et al., 2011, 2013), and these events likely result in enhanced mTORC1 activity given that MSTN signaling abrogates Akt activation (Morissette et al., 2009). As an interesting side note, Potts et al. (2017) recently used mass spectrometry-based phosphoproteomic analyses to demonstrate that over 600 phosphorylation events occur in rodent skeletal muscle 1-h following maximal-intensity contractions, and bioinformatics indicated that this phosphorylation signature was largely due to increased mTORC1 activity. Hence, beyond upregulating MPS, enhanced mTORC1 signaling hours following resistance exercise likely facilitates other physiological adaptations in skeletal muscle.

While mTORC1 is the hub that regulates MPS, there are multiple systems that regulate skeletal muscle proteolysis including (Pasiakos and Carbone, 2014; Tipton et al., 2018): (a) the calcium-dependent calpain system which liberates myofibrillar proteins from sarcomeric Z-lines, (b) the autophagy-lysosomal system which degrades cellular organelles as well as myofibrillar proteins, (c) the caspase system which cleaves myofibrillar proteins into smaller fragments, and (d) the ubiquitin-proteasome system (UPS) which uses E1/E2/E3 enzymes to poly-ubiquinate myofibril fragments and degrade these proteins into individual amino acids via the 26S proteasome. Proteolysis rates are likely influenced by a combination of these systems, and several human and rodent studies have reported biomarkers in each system are dynamically altered in response to acute and chronic resistance exercise training (Louis et al., 2007; Kerksick et al., 2010, 2013; Dalbo et al., 2013; Kwon et al., 2015; Stefanetti et al., 2015; Mobley et al., 2018b). Interestingly, rodent and *in vitro* studies have also demonstrated that inhibiting autophagy and UPS reduces skeletal muscle mass (Masiero and Sandri, 2010) and promotes myotube atrophy (Chandler et al., 2017), respectively, which suggests proteolytic mechanisms are seemingly obligatory for muscle mass maintenance. Mechanisms aside, human studies suggest: (a) chronic resistance exercise training increases MyoPS and MPS while reducing MPB in the post-absorptive state (Reidy et al., 2017a) and (b) a resistance exercise bout significantly elevates postabsorptive, post-exercise muscle proteolysis rates in the trained and untrained state, although the magnitude and duration of this increase is lower in the trained state (Phillips et al., 1999).

Compelling associations in humans have led to a general consensus that satellite cell-mediated myonuclear addition occurs during periods of resistance exercise training. For instance, numerous studies have used immunohistochemical staining techniques to demonstrate that satellite cell counts increase in response to one bout (Crameri et al., 2004; O’Reilly et al., 2008; Walker et al., 2012; Bellamy et al., 2014; Nederveen et al., 2015) and weeks of resistance exercise training (Kadi et al., 2004; Petrella et al., 2008; Verdijk et al., 2014; Reidy et al., 2017b). Many of these chronic training studies also reported myonuclear number concomitantly increases with satellite cell number (Petrella et al., 2008; Mobley et al., 2017; Reidy et al., 2017b). Such observations have led to a widespread hypothesis that satellite cell-mediated myonuclear addition supports fCSA increases during resistance exercise training. In fact it has been estimated that a 26% increase in fCSA can be achieved through training-induced alterations protein turnover (i.e., ↑ MPS and ↓ MPB), whereas satellite cell-mediated myonuclear addition occurs thereafter to maintain an adequate sarcoplasmic volume:myonucleus ratio and facilitate further hypertrophy (Kadi et al., 2004; Reidy et al., 2017b). This concept suggesting a myonucleus regulates a finite sarcoplasmic area is termed the myonuclear domain theory (Cheek et al., 1971; Hall and Ralston, 1989; Allen et al., 1999), and is discussed in greater detail below.

## Definition of Low Versus High Skeletal Muscle Hypertrophic Responders

It is important to highlight how different studies have defined high versus low skeletal muscle hypertrophic responders to resistance exercise training (summarized in **Table 1**).

**Table 1:** Studies clustering low versus high skeletal muscle hypertrophic responders.

Study (year)	Training summary	Criterion measure	Findings
Bamman et al., 2007	16 weeks of full body RT (3 d/wk) in younger and older males and females	Types I and II muscle fCSA changes	LR (*n* = 17): -16 μm^2^ HR (*n* = 17): +2,475 μm^2^
Davidsen et al., 2011	12 weeks of full body RT (5 d/wk) in college-aged males	Combination of histological, strength and DXA LBM changes	LR: ∼1.2 kg increase in DXA LBM HR: ∼4.5 kg increase in DXA LBM
Stec et al., 2016	4 weeks of full body RT (3 d/wk) in older males (60–75 years old)	Percent change in type II fCSA	LR (*n* = 17): -7% HR (*n* = 6): +83%
Ogasawara et al., 2016	12 weeks of leg extensor and curl training (3 d/wk) in college-aged males	Upper leg muscle size assessment via MRI	LR (*n* = 5): no increase in leg muscle size HR (*n* = 5): ∼20% increase in leg muscle size
Mobley et al., 2018a	12 weeks of full body RT (3 d/wk) in college-aged males	VL thickness (ultrasound)	LR (*n* = 17): 4% increase HR (*n* = 21): 30% increase

*LR, low responder; HR, high responder; DXA LBM, lean body mass assessed by dual X-ray absorptiometry; fCSA, fiber cross sectional area; MRI, magnetic resonance imaging; RT, resistance exercise training; VL, vastus lateralis.*

Notably, each study in **Table 1** has used different criterion variables to generate response cohorts. For instance two studies generated cohorts based upon pre- to post-training changes in fCSA (Bamman et al., 2007; Stec et al., 2016), whereas two other studies allocated muscle imaging techniques (Ogasawara et al., 2016; Mobley et al., 2018a), and one study used a combination of metrics (e.g., fCSA and whole-body lean tissue mass changes) (Davidsen et al., 2011). All of the training interventions also differed in training modality, duration, and frequency. Further, three of the aforementioned studies examined college-aged males (Davidsen et al., 2011; Ogasawara et al., 2016; Mobley et al., 2018a), one study examined older males (Stec et al., 2016), and the landmark study by Bamman et al. (2007) examined younger and older subjects from both sexes. These comparative differences aside, low responders typically experience little to no change in skeletal muscle hypertrophic indices (i.e., no appreciable changes in quadriceps volume or fCSA, ∼4% increase in vastus lateralis muscle thickness, or ∼0.5–1.2 kg increase in whole-body lean tissue mass). These average gains in low responders, while seemingly marginal, are still significantly different from pre-training levels when considering increases in VL thickness and whole-body lean tissue mass, and it is notable that Franchi et al. (2018a) have recently suggested even small changes in VL thickness can account for appreciable increases in muscle volume. Relative to these changes in low responders, however, high responders experience much more impressive increases in these metrics (i.e., 20% increase in quadriceps volume, ∼83% increase in fCSA, 30% increase in vastus lateralis muscle thickness, or ∼2.2–4.5 kg increase in whole-body lean tissue mass).

## Training-Induced Ribosome Biogenesis Is Greater in High Versus Low Responders

Ribosome biogenesis involves new ribosome formation through an increase in nucleolar transcriptional activity (Chaillou et al., 2014). Specifically, 5S and 45S ribosomal DNA (rDNA) repeats are enriched in the nucleolus, and an up-regulation in ribosome biogenesis occurs through increases in 5S ribosomal RNA (rRNA) transcription via RNA polymerase-III (Pol-III) activity as well as increases in 45S pre-rRNA via RNA Pol-I activity. Following 45S rRNA transcription, small nucleolar ribonucleoproteins (snoRNPs) cleave 45S pre-rRNA to form mature 18S, 5.8S, and 28S rRNAs. Thereafter, the formation of mature 60S and 40S ribosomal subunits is catalyzed by enzymes that assemble ribosomal proteins with the 5S/5.8S/18S/28S rRNAs as well as enzymes that facilitate ribosome export from the nucleus.

Given that ribosomes catalyze MyoPS and MPS, and repetitive post-exercise increases in these synthesis rates likely facilitate muscle growth, an increase in muscle fiber ribosome content during periods of resistance exercise training is seemingly advantageous for skeletal muscle hypertrophy. Akin to the myonuclear domain theory, ribosomes may regulate MyoPS and MPS in a finite area of the sarcoplasm. However, unlike the myonuclear domain theory which has been posited through observations involving nuclear staining methods and conventional light or immunofluorescent microscopy, empirically testing a ribosome domain theory is extraordinarily challenging given that ribosomes are remarkably small macromolecules (∼30 nm diameter) and are dispersed throughout the cell body. It is possible to detect skeletal muscle ribosomes through high-resolution transmission electron microscopy with appropriate gold-conjugated antibody labeling techniques (Gauthier and Mason-Savas, 1993). However, providing an accurate ribosome count per muscle fiber using this method would be laborious and has not been attempted. An *in situ* hybridization (ISH) method for 28S rRNA particle detection using conventional microscopy has been published using complimentary ^35^S-cRNA probes (Habets et al., 1999). These authors were able to visualize 28S rRNA (and presumably ribosome) particles within individual rat muscle fibers, and noted that smaller type I fibers paradoxically presented a fivefold to sixfold greater particle count relative to larger type II fibers. This method holds promise in terms of elucidating a potential ribosome domain, albeit this method or comparable methods (e.g., fluorescent *in situ* hybridization) have not been performed to track muscle fiber 28S rRNA particle changes during resistance exercise training.

A surrogate method that is commonly used for determining relative ribosome content includes assessing total RNA content per unit of wet muscle mass. This assumption is based upon 85% of total RNA existing as rRNA (Zak et al., 1967). Thus, increases in total RNA are likely reflective of rRNA increases, and rRNA increases are likely indicative of increases in ribosome content. Several studies using this method have reported inter-individual responses in ribosome biogenesis are related to differential hypertrophic responses to resistance exercise training. For instance, Figueiredo et al. (2015) examined younger, college-aged males and reported a high positive correlation existed between changes in quadriceps CSA and fold-change in ribosome content following 8 weeks of resistance exercise training (*r* = 0.72, *p* < 0.05). Bamman’s laboratory (Stec et al., 2016) subsequently reported that ribosome content increased ∼30% in older males (60–75 years old) that were high responders to 4 weeks of resistance exercise training (+83% type II fCSA), whereas no significant changes in ribosome content or fCSA were observed in low responders. Our laboratory reported similar findings in younger, college-aged males in that those experiencing robust increases in vastus lateralis (VL) muscle thickness (+30%; high responders) also experienced a 32% increase (*p* < 0.001) in muscle ribosome content following 12 weeks of resistance exercise training (Mobley et al., 2018a). Conversely, low responders experienced a small but significant increase in VL muscle thickness (+4%) coupled with an 8% non-significant increase in ribosome content following training (*p* = 0.25). Interestingly, Brook et al. (2017) recently employed the D_2_O tracer method to examine the rate of ribosome biogenesis during 6 weeks of resistance exercise training in college-aged males. These authors reported: (a) basal ribosome synthesis rates were ∼0.8% per day, whereas synthesis rates increased during the training period to ∼1.7% per day, and (b) increased basal ribosome biogenesis rates during training were significantly correlated to increased basal MPS rates (*r* = 0.57, *p* < 0.01). Additionally, other human training studies have similarly observed that rRNA increases parallel increases in hypertrophic indices following weeks of resistance exercise training (Kadi et al., 2004; Reidy et al., 2017a). Hence, these studies collectively demonstrate that the degree of ribosome biogenesis is associated with the degree of muscle hypertrophy during resistance exercise training and, according to the recent data from Atherton’s group, an increase in ribosome content parallels increases in basal MPS rates during training periods.

Beyond these human studies, mechanistic *in vitro* studies have similarly suggested ribosome biogenesis is critical for myotube growth. For instance, Nader et al. (2005) reported that increases in ribosome content paralleled cell growth in 20% serum-stimulated rat L6-derived myotubes. Stec et al. (2016) subsequently replicated these findings with 20% serum stimulation of primary human-derived myotubes, albeit pharmacological Pol-I inhibition completely abrogated both ribosome biogenesis and cell growth. In rats, recent evidence suggests the degree of synergist ablation-induced plantaris hypertrophy parallels proportional increases in ribosome content (Nakada et al., 2016). Our laboratory (Mobley et al., 2016, 2018b; Roberts et al., 2016) and others (West et al., 2016; Brook et al., 2017) have also reported that acute and chronic resistance exercise training models in rats increase indices of ribosome biogenesis and ribosome content, respectively.

Collectively, these *in vitro*, rodent, and human studies provide consistent evidence that the degree of ribosome biogenesis during a hypertrophic stimulus is associated with the degree of muscle growth that occurs therein. Notwithstanding, outstanding research questions remain to be investigated. For instance, it would be insightful to determine whether 5S and/or 45S ribosomal DNA (rDNA) copy number is greater in high versus low responders. To this end, inter-individual differences in rDNA copy number exist in rodents and humans (Wang and Lemos, 2017), although no publication to our knowledge has reported the relationship between rDNA copy number and markers ribosome biogenesis following one or multiple bouts of resistance exercise training. Additionally, determining whether rDNA copy number differs between high versus low responders remains to be explored. Examining these relationships holds exciting promise in determining how inter-individual variation in rDNA copy number affects resistance exercise training-induced changes in muscle size.

## Satellite Cell-Mediated Myonuclear Addition May Dictate the Hypertrophic Response to Resistance Exercise Training

Satellite cell-mediated myonuclear accretion seemingly occurs during longer-term periods of resistance exercise training, and high responders may experience this phenomenon to a greater extent relative to low responders due to robust increases in satellite cell number and fusion potential. Data from Bamman’s laboratory supports this paradigm in that pre- and post-training satellite cell counts were greater in high versus low hypertrophic responders following 16 weeks of training (Petrella et al., 2008). Bellamy et al. (2014) also observed that subjects experiencing the greatest increases in satellite cell proliferation 72 h following a naïve training bout experienced the greatest increases in quadriceps volume changes following 16 weeks of subsequent resistance exercise training. Additionally, other laboratories have observed increases in satellite cell number following resistance exercise training is associated with increased fCSA values (Kadi et al., 2004; Verdijk et al., 2014; Reidy et al., 2017b). These findings have led to a general consensus that satellite cell-mediated myonuclear addition is likely an involved mechanism in promoting skeletal muscle hypertrophy, and this hypothesis is further supported by studies which have illustrated high associations exist between fCSA and myonuclear number (Kadi et al., 1999; Hikida et al., 2000). Notably, only our study and Petrella et al. (2008) sought to determine if chronic resistance exercise training differentially affected satellite cell number between low versus high skeletal muscle hypertrophic responders. Contrary to the findings of Petrella et al. (2008), as well as the hypothesis that increases in satellite cells are obligatory for resistance exercise training-induced muscle hypertrophy, we reported that training-induced increases in satellite cell number and increases in types I and II fiber myonuclear number were similar between high versus low responders following 12 weeks of resistance exercise training (Mobley et al., 2018a). While it is difficult to reconcile why our data differed from the aforementioned study by Petrella et al. (2008), critical differences between these studies should be noted. First, several different staining methods exist for satellite cell quantification (e.g., Pax7 versus NCAM staining, as well as DAB versus fluorescent imaging) (Lindstrom and Thornell, 2009). Notably, our study identified satellite cells as Pax7(FITC)+/DAPI+ cell bodies using immunofluorescent microscopy, and Petrella et al. (2008) identified satellite cells as NCAM(DAB)+ cell bodies using light microscopy. FITC quantification can yield a high level of autofluorescence (*unpublished observations*), and this methodological difference between studies may have artificially inflated our satellite cell counts relative to Petrella et al. (2008). Second, response clusters examined by Petrella et al. (2008) included college-aged and older (60–75 years old) subjects from both sexes, whereas our study consisted of only college-aged males. It is also notable that an earlier publication by Bamman’s group (Petrella et al., 2006) examined many of the same subjects contained in the Petrella et al. (2008) paper, and the authors noted college-aged males (not older males or females) experienced the most robust increases in fCSA, satellite cell counts and myonuclear addition following training. Alternatively stated, it appears that college-aged males (i.e., generally high responders) experience greater increases in satellite cell counts and satellite cell-mediated myonuclear addition during periods of resistance exercise training relative to older subjects or females (i.e., generally low responders).

Regarding the role of satellite cells during periods of resistance training or overload, there is compelling evidence in rodents which challenge the necessity of satellite cell-mediated myonuclear addition for skeletal muscle hypertrophy. In this regard, a landmark study by McCarthy et al. (2011) used adult, female Pax7-DTA mice to ablate >90% of satellite cells via intraperitoneal tamoxifen injections. Notably, synergist ablation-induced plantaris hypertrophy doubled in both tamoxifen- and vehicle-treated mice following 2 weeks of overload, and hypertrophy in tamoxifen-treated mice still occurred in the absence of myonuclear addition. Indeed, these findings have been challenged by another laboratory suggesting that tamoxifen-induced depletion of satellite cells in adult female Pax7-DTA mice does indeed prevent synergist ablation-induced plantaris and EDL myofiber hypertrophy (Egner et al., 2016). Nevertheless, at least in murine models, these conflicting reports do not provide conclusive evidence suggesting satellite cell-mediated myonuclear addition is obligatory for overload-induced, supraphysiological hypertrophy. It is also noteworthy that, in humans, while it has been posited that myonuclear accretion is needed to offset domain expansion and contribute to fCSA increases >26% (Kadi et al., 2004), some studies contradict this hypothesis. For instance, increases in satellite cell number and myonuclear addition have been shown to occur in the absence of fCSA increases following 12 weeks of resistance exercise training (Mackey et al., 2007). A recent study also suggests myonuclear domain expansion does not occur during or following 12 weeks of resistance exercise training in college-aged men despite observed increases in fCSA, satellite cell number, and myonuclear number (Snijders et al., 2016). Damas et al. (2018) also reported that type II fiber satellite cell content significantly increased in college-aged men 48 h following a naïve training bout, as well as prior to and 48 h following training bouts that occurred 3- and 10 weeks into resistance exercise training. However, while the authors observed a significant increase in type II fCSA by week 10 of training, virtually no change occurred in type II fiber myonuclear number. Notwithstanding, an overwhelming majority of evidence from human studies does suggest that resistance exercise training increases satellite cell number acutely and chronically, and these findings collectively underscore the important role that satellite cells likely have in the adaptive response.

## Select Skeletal microRnas May Influence the Hypertrophic Response to Resistance Exercise Training Via IGF-1 Induction

microRNAs (miRs) are small non-coding RNA molecules that are ∼20 nucleotides in length and function to inhibit the translation of select mRNAs in a sequence-specific fashion. Since the discovery of miRs in the 1990s, several research groups have studied the skeletal muscle miR response to exercise training (Silva et al., 2017). Interestingly, there is evidence suggesting select skeletal muscle miRs may be differentially expressed between high versus low responders during periods of resistance exercise training. Davidsen et al. (2011) examined 21 mature skeletal muscle miRNAs prior to and following 12 weeks of resistance exercise training in college-aged men, and reported that miR-378, miR-29a, and miR-26a were downregulated in low responders and unchanged in high responders, whereas miR-451 was upregulated only in low responders. The authors also reported skeletal muscle IGF-1 mRNA levels were only upregulated in the high responders, and bioinformatics suggested the observed miR signature in low responders may be a compensatory mechanism attempting to activate genes related to growth factor signaling. Interestingly, these data partially replicated Bamman’s original responder paper given high responders in both studies experienced significant increases in basal skeletal muscle IGF-1 mRNA expression levels following training (Bamman et al., 2007). It is also notable that muscle miR levels are dynamically altered during supraphysiological plantaris hypertrophy following synergist ablation in mice, and this mechanism may also act to upregulate IGF-1 mRNA expression. To this end, McCarthy and Esser (2007) reported that 7 days of synergist ablation increased plantaris mass by 45% and down-regulated plantaris miR-1 and miR-133a levels by ∼50%, and these authors speculated that a down-regulation in miR-1 may serve to up-regulate IGF-1 levels during overload given that the seed region of miR-1 targets IGF-1 mRNA. Taken together, skeletal muscle miR expression patterns in high responders during training, or in mice experiencing supraphysiological hypertrophy, may lead to an upregulation in IGF-1 mRNA levels which acts to further enhance anabolic signaling. However, this mechanism is highly speculative and should be researched further.

Differential muscle mIR profiles in high versus low responders has also been reported by Ogasawara et al. (2016) who demonstrated over 100 mIRs were altered following 12 weeks of resistance exercise training in college-aged men, and miR-30d-5p and miR-376a-3p were differentially expressed between high versus low responders 3 h following a training bout as well as after 6 weeks of training. However, potential mRNA targets these miRs could have affected were not provided. Thus, more investigative *in vitro* work (e.g., mIR transfection experiments) is needed in order to determine if the aforementioned miR candidates affect muscle fiber hypertrophy.

## Skeletal Muscle Androgen Receptor Induction May Delineate the Hypertrophic Response to Resistance Exercise Training

Testosterone and other androgens exert their physiological effects on different tissues through binding to androgen receptors localized in the sarcoplasm. Upon ligand binding, androgen receptors translocate to the nucleus to act as a transcription factor and alter the mRNA expression of hundreds to thousands of genes (Jiang et al., 2009). Given that enhanced androgen receptor signaling in skeletal muscle through the administration of anabolic steroids has been linked to increased satellite cell proliferation (Sinha-Hikim et al., 2002, 2003) and MPS (Griggs et al., 1989; Ferrando et al., 1998), a high level of enthusiasm exists regarding the hypertrophic effects of this pathway. Interestingly, two studies have demonstrated that changes in skeletal muscle androgen receptor protein content correlate with increases in skeletal muscle hypertrophy. Ahtiainen et al. (2011) reported skeletal muscle androgen receptor protein increases correlated with fCSA and lean body mass increases in younger and older men following 21 weeks of resistance exercise training. Mitchell et al. (2013) subsequently reported skeletal muscle androgen receptor protein increases, not serum testosterone levels, correlated with fCSA increases following 12 weeks of resistance training college-aged men. However, we recently reported (Mobley et al., 2018a) that high and low responders similarly exhibit a downregulation in androgen receptor protein levels with training. We speculate that this downregulation with training was potentially due to negative feedback; specifically, if androgen signaling is enhanced with resistance exercise training then receptor levels would likely be down-regulated in order to prevent excessive signaling from occurring. Additionally, the Ahtiainen et al. (2011) and Mitchell et al. (2013) studies associated androgen receptor protein changes with fCSA and/or lean body mass changes (not VL thickness changes) which could have led to discordant findings. Notwithstanding, multiple studies suggest increases in androgen receptor protein content may promote further increases in skeletal muscle hypertrophy during resistance exercise training, and more studies are needed in order validate this potential mechanism.

## Do “Favorable” Genetics Delineate Skeletal Muscle Hypertrophic Response Clusters to Resistance Exercise Training?

Heritability studies have estimated ∼50% exercise training adaptations are influenced by genetics (Mann et al., 2014), and it is widely speculated that “favorable” genetics facilitate optimal training adaptations. A well-documented case study that has commonly been cited as showing “favorable” genetics promote a muscular phenotype involves a child with a homozygous MSTN mutation (Schuelke et al., 2004). Notably, this subject presented an exceptionally muscular phenotype for his age, and the MSTN mutation was shown to result in functionally deficient protein. However, it is highly unlikely that such rare mutations exist in the upper quartile of individuals that are high responders to resistance exercise training, and “favorable” genetics likely includes a combination of numerous polymorphisms.

A powerful approach that does possess the potential to decipher if a combination of polymorphisms associates with differential hypertrophic responses to resistance exercise training are genome-wide association studies (GWAS). GWAS utilizes DNA hybridization arrays or next generation sequencing to interrogate thousands to millions of common single nucleotide polymorphisms (SNPs), insertion-deletion alleles, or genomic repeat alleles. As a contextual example, Bouchard et al. (2011) used GWAS to examine over 320,000 SNP candidates related to the VO_2_max responses in HERITAGE study participants. The authors reported that 21 SNP candidates accounted for 49% of the shared variance in VO_2_max changes, and subjects who carried ≤9 of these favorable alleles improved their VO_2_max by 221 mL/min whereas those who carried ≥19 of these alleles improved their VO_2_max by 604 mL/min. No GWAS study has been carried out to determine if a combination of SNP candidates might share variance with hypertrophic outcomes following resistance exercise training; however, in principal, Bouchard’s data demonstrates that a combination of variant alleles are likely responsible for some of the divergence of this trait.

A targeted approach commonly used to examine if genetics is related to an exercise phenotype includes restriction enzyme- or TaqMan-based polymerase chain reaction techniques. Two very well-studied SNP candidates related to exercise phenotypes include the ACE I/D and ACTN3 R577X genotypes (Guth and Roth, 2013). Various studies have suggested that the ACE I/I genotype is more common in endurance athletes (Puthucheary et al., 2011). However, this SNP has been reported to not affect the degree of muscle hypertrophy following 10 weeks of knee extensor training in older men and women (Charbonneau et al., 2008). The ACTN3 R577X genotype may affect strength outcomes following resistance exercise training. For instance, Clarkson et al. (2005) reported females with the XX genotype experienced significant increases in strength following 12 weeks of upper body resistance exercise training compared to females with the RR genotype (69% versus 56%, respectively). However, all genotypes experienced similar increases in muscle size following training (∼2.5%), and only 2% of reported strength gains after training were attributable to the ACTN3 R577X genotype. Examples of other studies using these targeted approaches to identify SNPs associated with differential hypertrophic responses to resistance exercise training include the following:

(a) Young men with the bradykinin type 2 receptor (B2BRK) -9/-9 genotype (∼21% of subjects) experienced an 8.5% increase in triceps brachii muscle thickness following 6 weeks of resistance exercise training, whereas those with the +9/-9 or +9/+9 genotype experienced a significantly lower degree of hypertrophy (4.7% combined) (Popadic Gacesa et al., 2012).
(b) College-aged Chinese men possessing one or two T alleles for the MSTN A55T genotype (∼14% of subjects) experienced a 12.6% increase in biceps muscle thickness following 8 weeks of resistance exercise training, whereas those with the AA genotype experienced a significantly lower degree of hypertrophy (8.2%) (Li et al., 2014). These authors also examined another MSTN SNP in these same subjects and reported that those possessing one R allele for the MSTN K135R genotype (∼6% of subjects) experienced 12.9% and 9.1% increases in biceps and quadriceps muscle thicknesses, respectively, following training whereas those with the KK genotype experienced a significantly lower degree of hypertrophy (8.6% and 3.9%, respectively).
(c) Older Caucasian men and women (average age ∼70 years old) possessing at least one cytosine adenine dinucleotide repeat in the IGF-1 promoter region (∼87% of subjects) experienced ∼130 mL increase in quadriceps muscle volume following 10 weeks of knee extensor resistance exercise training, whereas homozygotes lacking this allele experienced a lower degree of hypertrophy (∼95 mL) (*p* = 0.08 between genotypes) (Kostek et al., 2005).

Collectively, these studies illustrate genetic variation is likely responsible for some of the differential hypertrophic response to resistance exercise training. However, magnitude differences for hypertrophic outcomes between genotypes in these studies are not nearly as impressive as what has been reported for high versus low responders (**Table 1**). Additionally, as Bouchard et al. (2011) reported with HERITAGE participants, a combination different of SNPs/insertions-deletions/tandem repeats are likely prevalent in high versus low skeletal muscle hypertrophic responders. Thus, replicating Bouchard’s GWAS approach in a large training cohort is needed to gain greater insight as to how genetic variation contributes to differential hypertrophy responses to resistance exercise training.

## Pre-Training Fiber Type Composition Does Not Likely Delineate Response Clusters to Resistance Exercise Training

Elite strength/power athletes possess a high proportion of fast-twitch/type II muscle fibers (∼60%) (Tesch et al., 1984; Trappe et al., 2015), whereas elite endurance athletes possess a high proportion of slow-twitch/type I muscle fibers (∼70%) (Ingjer, 1979). These observations have led to a general consensus that individuals possessing a high proportion of type II muscle fibers are predisposed to be talented strength/power athletes, whereas those possessing a high proportion of type I fibers are predisposed to be talented endurance athletes. In line with this rationale, it is possible that untrained individuals with a greater proportion of type II fibers may experience a greater degree of muscle hypertrophy during periods of resistance exercise training relative to individuals with a high proportion of type I fibers given that type II fibers are typically larger than type I fibers (∼5,000 μm^2^ versus ∼4000 μm^2^). However, a recent review cites multiple lines of evidence suggesting type I and II fCSAs similarly increase with higher volume resistance exercise training, and that the “growth potential” of both fiber types appear to be similar (Ogborn and Schoenfeld, 2014). It is also notable that our cluster analysis (Mobley et al., 2018a) as well as two of Bamman’s cluster analysis papers (Bamman et al., 2007; Stec et al., 2016) suggest pre-training type I/II fiber type distribution patterns do not differ between low versus high responders. Taken together these studies suggest pre-training fiber type likely does not appreciably dictate the hypertrophic response potential to resistance exercise training.

## Future Research Directions Examining Factors Which May Contribute to Differential Hypertrophy Responses to Resistance Exercise Training

The remainder of this article presents factors which could theoretically distinguish high versus low responders. Importantly, little to no data supports these factors to differentially affect the hypertrophic response to resistance exercise training and, as such, these relationships should be further examined.

### Does Connective Tissue Limit Skeletal Muscle Growth in Response to Resistance Exercise Training?

It is logical that connective tissue thickness and malleability potential may limit skeletal muscle growth, although no human studies have directly illustrated this concept. A recent transcriptomic interrogation in humans suggests combined endurance and resistance exercise training chronically upregulates the mRNA expression of skeletal muscle genes related to collagen synthesis and extracellular matrix remodeling (e.g., MXRA5, COL1A1, COL3A1, COL4A1) (Hjorth et al., 2015). Notwithstanding, the authors did not determine if the fold-change magnitude in these genes were associated with hypertrophic outcomes. There is stronger evidence in animals suggesting connective tissue components limit skeletal and cardiac muscle growth. Perhaps the strongest evidence suggesting connective tissue acts as a governor of myocyte growth is in a study in pigs whereby the surgical removal of pericardial sheath elicited a rapid 18% increase in cardiac hypertrophy 14–21 days post-surgery (Hammond et al., 1992). However, this was a secondary outcome of the study and the authors did not elaborate on the potential significance of these findings relative to skeletal muscle physiology. Impaired overload-induced hypertrophy has been reported in IL-6^-/-^ versus wild-type mice, and this mal-adaptation was associated with a significantly greater accumulation of hydroxyproline and procollagen-1 mRNA (White et al., 2009). Fry et al. (2014) also reported that satellite cell depletion in the Pax7-DTA mouse line reduced plantaris type IIa/x fCSA increases following 8 weeks of synergist ablation relative to vehicle-treated mice, and noted satellite cell depletion significantly increased fibroblast and collagen accumulation around individual muscle fibers. Interestingly, these authors also used intricate co-culturing methods to demonstrate primary isolated satellite cells down-regulated mRNA levels of collagen-related genes in fibroblasts. In explaining the significance of these findings, the authors posited the enhanced deposition of collagen in the extracellular matrix may have constricted synergist ablation-induced hypertrophy in satellite cell-depleted mice, and satellite cells act to offset this phenomenon by secreting miR-containing vesicles which target/downregulate collagen-related genes in fibroblasts. This hypothesis was supported through a follow-up study by this research group which reported satellite cell depletion prior to synergist ablation increased skeletal muscle collagen deposition in the extracellular matrix and impaired muscle hypertrophy 8 weeks following synergist ablation, although inducing satellite cell depletion 1 week into synergist ablation rescued this effect (Fry et al., 2017). Therefore, if high responders do benefit from a heightened satellite cell proliferation response during training then this may be due, in part, to the “supporting role” that satellite cells have on promoting extracellular matrix adaptations (Murach et al., 2018). These interesting observations in animals aside, future studies are needed to determine if changes in muscle collagen content, and/or mRNAs or miRs related to extracellular matrix remodeling differentiate high versus low responders.

### Does the Inflammatory Response to Resistance Exercise Training Limit Skeletal Muscle Growth?

Heightened inflammatory states during cancer/cachexia, infections, and extensive tissue trauma have been linked to skeletal muscle catabolism (Bistrian et al., 1992). From a mechanistic perspective, pro-inflammatory cytokines (e.g., TNF-α and IL-1β) upregulate proteolytic activity in skeletal muscle (Zamir et al., 1992; De Larichaudy et al., 2012; Wang et al., 2014). While IL-6 is not entirely pro-inflammatory and has several pleiotropic roles in skeletal muscle (Munoz-Canoves et al., 2013), rodent (Haddad et al., 2005), and human data (Raj et al., 2008) suggest chronic elevations in IL-6 upregulate skeletal muscle proteolysis as well. Prostaglandin PGE_2_ has also been shown to stimulate IL-6 mRNA expression in order to further upregulate inflammatory signaling (Standley et al., 2013), and the mRNAs for TNF-α and IL-6 as well as PGE_2_ levels in skeletal muscle have all been shown to be upregulated during the post-exercise period following a single resistance exercise bout (Trappe et al., 2001; Louis et al., 2007). Thus, for reasons listed above, it is conceivable that individuals who fail to down-regulate these markers between training bouts may experience a stagnation in muscle growth.

Indeed, there is precedence suggesting a differential inflammatory mRNA expression signature in skeletal muscle is related to the individual hypertrophic responsiveness to resistance exercise training. For instance, Raue et al. (2012) highlighted data demonstrating the fold-change of two-inflammatory-related mRNAs (TNFRSF12A and NFKBIA) 4 h following a naïve resistance exercise bout were positively and negatively correlated, respectively, with increases in quadriceps muscle CSA following 12 weeks of resistance exercise training. Additionally, while Dennis et al. (2009) demonstrated that certain skeletal muscle mRNAs associated with inflammation were associated strength (not hypertrophy) in older subjects following 12 weeks of resistance exercise training (e.g., IL-1β, IL-1β receptor agonist and IL-10 mRNA decreases all strongly correlated with strength gains), Thalacker-Mercer et al. (2013) subsequently reported several mRNAs related to the NF-κB inflammatory signaling cascade were down-regulated in high versus low responders following a 16-week training protocol. Similar to these findings, we recently reported that IL-1β mRNA was significantly down-regulated in high versus low responders following 12 weeks of training (Mobley et al., 2018a).

There is counterevidence, however, suggesting heightened post-exercise inflammation actually facilitates skeletal muscle hypertrophy. For instance, post-exercise increases in prostaglandin PGE_2_ and PGF_2α_ stimulate post-exercise increases in muscle protein synthesis (Trappe et al., 2001, 2002). Recent *in vitro* evidence also suggests myotubes treated with IL-6 upregulate mTORC1 signaling and myotube protein synthesis (Gao et al., 2017). Further confounding this issue is evidence suggesting the inhibition of inflammatory signaling via NSAIDs during periods of resistance exercise training does not affect hypertrophic outcomes. For instance, the daily consumption of over-the-counter doses of ibuprofen, which inhibits muscle prostaglandin synthesis, was reported to have no effects on muscle thickness increases in college-aged subjects over a 6-week resistance exercise training period (Krentz et al., 2008). Similar outcomes have also been reported in older adults (∼65 years old) following 12 weeks of lower-body training (Trappe et al., 2011). It is also notable we reported certain aspects of inflammatory signaling do not differ between high and low responder cohorts (i.e., serum levels of IL-6 as well as skeletal muscle phosphorylated p65/NF-κB, IL-6 mRNA, and TNF-α mRNA) (Mobley et al., 2018a). To summarize, while preliminary evidence suggests that select mRNAs related to inflammatory signaling may be differentially expressed in low versus high responders, there is not enough experimental evidence to suggest low responders exist in a heightened inflammatory state during training periods. Notwithstanding, potential relationships between differential hypertrophic responses to resistance exercise training and inflammatory signaling should be further explored in other subject populations susceptible to increased inflammation (e.g., older subjects) given the theoretical rationale suggesting heightened inflammation favors skeletal muscle catabolism.

### Is There a Relationship Between Mitochondrial Characteristics and Hypertrophic Responders?

Differences in muscle mitochondrial function and/or volume may also exist between high versus low responders. Notably, it has been estimated that upward of four ATP molecules are required per peptide bond synthesized (Stouthamer, 1973). It is therefore plausible that increases in mitochondrial function or volume are needed to sustain muscle growth during resistance exercise training due to the energy required for sarcoplasmic and myofibrillar protein accretion. Groennebaek and Vissing (2017) authored a recent review including 16 studies which examined how chronic “high load” resistance exercise training affected mitochondrial volume and function. These authors noted only two of these studies reported an increase in mitochondrial volume after 12 weeks of training while the other 14 studies reported no change or decreases. However, these authors did also note that three of the five chronic training studies that did measure function in permeabilized fibers reported improved indices of mitochondrial function (e.g., a tighter coupling of oxidative phosphorylation) (Pesta et al., 2011; Salvadego et al., 2013; Porter et al., 2015). Thus, resistance exercise training may generally increase mitochondrial function without affecting mitochondrial volume changes, and examining these phenomena in low versus high responders would provide greater insight as to whether there is mitochondrial involvement in differential hypertrophic responses. It should also be noted recent evidence suggests mechanisms regulating ribosome biogenesis and mitochondrial biogenesis may also be in direct opposition of one another (Gibbons et al., 2014). Specifically, these authors used advanced genomic sequencing and bioinformatics techniques to discover that mtDNA abundance, which is loosely associated with mitochondrial volume and also demonstrates a high degree of inter-individual variability like rDNA copy number, was significantly negatively associated with rDNA copy number. These data imply if high responders do possess a higher rDNA copy number (yet to be tested) then it is also possible that these same individuals would also have a lower mitochondrial volume relative to low responders. Hence, clarification is needed in delineating mitochondrial characteristics as well as rDNA copy number in high versus low responders.

### Do Hypertrophic Response Clusters Possess Differences in Vascular Properties?

Resistance exercise has been reported to upregulate the mRNA expression of the pro-angiogenic vascular endothelial growth factor (VEGF) hours following a single bout (Gavin et al., 2007), and this phenomenon likely participates in *de novo* skeletal muscle capillary formation reported with longer term training (McCall et al., 1996; Verdijk et al., 2016; Nederveen et al., 2017). While no literature to date has established that training-induced increases in capillary number is obligatory for fCSA increases, some studies have reported a tight coupling between fCSA increases and increases in capillary number per fiber following resistance exercise training (Verdijk et al., 2016; Nederveen et al., 2017). Additionally, Snijders et al. (2017) recently reported that older men with higher baseline capillary densities experienced greater increases in type II fCSA and satellite cell counts relative to individuals with lower capillary densities following 24 weeks of resistance exercise training. There is also evidence suggesting skeletal muscle capillary content and/or microvascular endothelial function may directly influence muscle fiber size. For instance, middle-aged to older sarcopenic subjects have been reported to possess a significantly lower skeletal muscle capillary content compared to age-matched non-sarcopenic counterparts (Prior et al., 2016), and the authors interpreted these findings to suggest transcapillary transport of nutrients, anabolic hormones, and oxygen to muscle is critical for muscle mass maintenance. This study parallels other evidence suggesting impaired endothelial function with aging reduces the anabolic response to amino acid feeding (Timmerman et al., 2012), and these authors similarly posited a reduction in skeletal muscle blood flow may contribute to sarcopenia due to a reduction in nutrient delivery. Thus, future research examining if high responders possess a higher capillary content or enhanced endothelial function compared to low responders is warranted, albeit the aforementioned Snijders et al. (2017) study is already beginning to establish that this relationship exists.

### Other Considerations

First, a major consideration regarding all of the aforementioned studies examining characteristics between high versus low responders is that these studies are age- and gender-biased toward college-aged males. Exceptions to this bias include the Bamman et al. (2007) publication which studied both younger and older subjects from both sexes (Bamman et al., 2007), and the 2016 publication from the same laboratory which studied older males (Stec et al., 2016). Hence, the aforementioned muscle biomarkers that associate with high and low responders should be viewed in this context, and more studies are needed to validate these targets in females and older populations.

Second, while this review was meant to be as comprehensive as possible on the topic, there are still other mechanisms to consider which may delineate skeletal muscle hypertrophic responses to resistance exercise training. For instance, while transcriptome-wide differences between low and high responders skeletal muscle hypertrophic responders has been reported as discussed above (Thalacker-Mercer et al., 2013), it is also notable that Raue et al. (2012) also performed transcriptome-wide profiling and reported that 661 genes which were affected by resistance exercise training were correlated to changes in muscle size and strength. Additionally, similar transcriptome-wide studies suggest select pre-training mRNAs and/or the fold-change induction in certain mRNAs correlate with changes in hypertrophic indices following resistance exercise training (Dennis et al., 2009; Phillips et al., 2013). Hence, these collective –omics-based data implicate that numerous intrinsic molecular signaling pathways, many of which are presumably unidentified and not mentioned herein, likely delineate high versus low hypertrophic responders.

Third, a critical extrinsic factor not discussed herein and deserves mentioning includes differences in dietary habits that exist between high versus low responders. A general consensus in the scientific literature is that higher protein, hypercaloric diets optimize skeletal muscle hypertrophy during periods of resistance exercise training (Volek et al., 2006; Campbell et al., 2007). Thus, one potential hypothesis could be that high responders may subscribe to these dietary practices more so than low responders. It is compelling, however, that our data (Mobley et al., 2018a) and Bamman’s data (Thalacker-Mercer et al., 2009) suggest self-reported caloric and protein intakes do not differ between high versus low responders. While this evidence is limited to two studies, these findings exclude the possibility that low responders could benefit from additional dietary protein and/or calories. In this regard, Reidy and Rasmussen (2016) compiled evidence from numerous studies (*n* = 95 total subjects) illustrating that there are both low and high skeletal muscle hypertrophic responders which may not experience added benefit to protein supplementation. Notwithstanding, designing studies to identify low responders during training and then feeding said participants a higher amount of protein or energy will provide more insight.

Finally, it is critical for the reader to appreciate that low skeletal muscle hypertrophic responders still (on average) observe beneficial training adaptations. Our recent study examining low responders, assessed via VL thickness changes, suggest that this group experienced significant increases in whole-body lean tissue mass (+2.2 kg versus +3.3 kg in high responders, *p* > 0.05) as well as lower body strength (+31 kg versus +39 kg in high responders, *p* > 0.05) following training (Mobley et al., 2018a). Further, and as mentioned earlier, there clear distinctions between study methodologies used to generate response clusters both from statistical (e.g., K-means cluster versus percentile rank) and methodological perspectives (e.g., clustering based upon fCSA versus VL thickness versus a combination of metrics). These between-study differences are clearly a limitation since single methodologies used to assess changes in muscle mass following training have been shown to poorly correlate (e.g., MRS-determined VL volume versus VL thickness assessed via ultrasound) (Franchi et al., 2018a). Moreover, there is criticism of statistical clustering methods used to identify responders versus non-responders following training interventions (Atkinson and Batterham, 2015). Additionally, these authors posited that “comparator” (or non-training) arms are typically lacking from studies which posit biomarkers that delineate low versus high exercise responders. Therefore, moving forward, the field should attempt to standardize the definition of low versus high skeletal muscle hypertrophic responders, and we posit that using multiple metrics (e.g., pre-to-post training changes in fCSA, lean tissue mass, and VL thickness) would be a more preferable approach compared to using one criterion clustering variable. Furthermore, implementing comparator arms in future studies will provide data regarding magnitude-based or statistical changes that occur in both low and high responders relative to a non-training group.

## Conclusion

**FIGURE 1:**
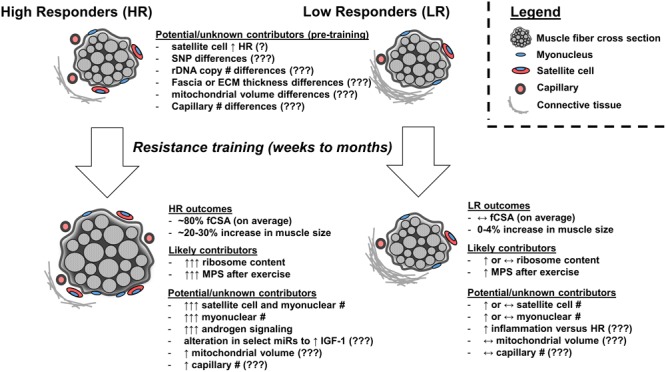
Factors which may or likely affect the skeletal muscle hypertrophic response to resistance exercise training. High skeletal muscle hypertrophic responders (HR) experience, on average, ∼80% in fiber cross sectional area (fCSA) or a 20–30% increase in muscle size following weeks to months of resistance exercise training. Low responders (LRs) experience virtually no change in fCSA or muscle size. Pre-training differences between clusters have included greater satellite cell number in high versus low responders; however, these findings have not replicated in other studies (indicated by ‘?’). Other speculative pre-training differences which may exist and need to be researched include connective tissue properties (e.g., fascia thickness, proteins related to extracellular matrix), genetic differences (e.g., multiple SNP candidates through GWAS), rDNA copy number differences, mitochondrial volume or function differences, or capillary differences (indicated by ‘???’). Following training, the observed phenotype in HR is influenced in part by superior increases in ribosome biogenesis and subsequent elevations in basal and post-exercise MPS. Limited evidence suggests HR experience superior training-induced increases in satellite cell number and satellite cell-mediated myonuclear addition as well as an altered micro RNA (miR) response to training to potentially enhance IGF-1 gene expression and increased androgen receptor protein content, although more research is needed to explore these areas. It is speculative whether HR experience superior increases in mitochondrial volume or capillary number following training relative to LR (indicated by ‘???’). It is also speculative whether LR experience a heightened inflammatory response to training (indicated by ‘???’).

Several intrinsic factors likely drive the hypertrophic response to resistance exercise training (summarized in **Figure 1**). There are human reports from several laboratories suggesting the degree of ribosome biogenesis during training associates with the degree of hypertrophy, and the importance of ribosome biogenesis in facilitating muscle hypertrophy is strengthened by numerous *in vitro* and rodent studies. Other factors which have been studied and may contribute to differential hypertrophic responses to resistance exercise training include: (a) a heightened capacity for satellite cell proliferation and satellite cell-mediated myonuclear addition, (b) differential expression patterns in select skeletal muscle miRs following acute bouts and chronic training, (c) elevated androgen receptor protein content in skeletal muscle, and (d) the presence of certain genomic SNP/insertion-deletion/repeat variants. However, these factors should be more thoroughly investigated given some of the sparse or conflicting data presented herein. Other intrinsic factors which we speculate may lead to differential hypertrophic responses to training include rDNA copy number, extracellular matrix and connective tissue properties, inflammatory signaling, mitochondrial characteristics, and/or microvascular characteristics.

Research identifying intrinsic factors that regulate differential hypertrophic responses to resistance exercise training will generate future research which examines if these factors can be modulated by altering extrinsic variables such as nutrition, exercise dosing, or recovery strategies. Importantly, these series of scientific conquests will ultimately improve our understanding of factors that optimize resistance exercise training adaptations, and such research will likely be useful for individuals seeking to apply this knowledge in a practical setting.

## Author Contributions

All authors listed have made a substantial, direct and intellectual contribution to the work, and approved it for publication.

## Conflict of Interest Statement

The authors declare that the research was conducted in the absence of any commercial or financial relationships that could be construed as a potential conflict of interest.
